# The current role of Active Surveillance in early prostate cancer

**Published:** 2009

**Authors:** R. P. Shrinivas, Deepak Dubey

**Affiliations:** Manipal Hospital, Rustam Bagh, Airport Road, Bangalore 560 017, India E-mail: drdeepakdubey@rediffmail.com

## COMMENT

The authors retrospectively analysed multicentric data, from four centers in Europe who participated in the screening arm of the European Randomized Study of Screening Prostate Cancer (ERSPC), diagnosed with prostate cancer and treated initially with Active Surveillance (AS).

Patients included for AS had been screen-detected for small, localized, well-differentiated prostate cancer, which satisfied the Prostate Cancer Research International: Active Surveillance (PRIAS) criteria for AS. Prostate-specific antigen [PSA] ≤ 10.0 ng/ml, PSA-density < 0.2 ng/ml per ml, Stage T_1C_/T_2_, Gleason score ≤ 3 + 3 = 6, ≤2 positive biopsy cores with no known positive lymph nodes or distant metastases).

Of the 988 men treated with AS, only 616 conformed to PRIAS criteria. The mean follow-up time was 4.35 years (range: 0–11.6 years). The calculated 10-year prostate cancer specific survival rate was 100% and the 10-year overall survival rate was 77%. This indicates that at the time of analysis, 563 patients were alive, of which 381 (68%) were continuing on AS and 182 underwent deferred active treatment.

Of the 419 (68%) men who continued AS, 30 had a PSA >10 ng/ml, 29 had PSA-DT < 3 years, 8 had both making them candidates for active therapy. Contrastingly, there were 110 out of 197 men (55.8%) who received deferred therapy despite a favorable PSA and PSA-DT. This switch over to active therapy was not influenced by a clinical or pathologic assessment in the form of DRE or rebiopsies, respectively.

The strength of the study was the size of the group, being multicentric along with a follow-up period and a large number of PSAs available. The authors conclude that screen-detected PCa that fits the current criteria for AS have a favorable PCa-specific prognosis. After the 10-year follow-up period, 100% of the patients survived their cancer but almost one-fourth died from other causes.

On the basis of the PSA characteristics of all 616 patients, the authors found that a small fraction (1.9%; 8 of 419) of the patients who remained untreated may be (or may have been) better candidates for active treatment, whereas 55.8% (110 of 197) of men who did receive active therapy were not obvious candidates for radical treatment. Factors such as anxiety, urologic complaints, or comorbidity information may have been more decisive, but these were not available in the study.

These results indicate that a significant number of patients on AS proceed to active deferred treatment in the absence of documented progression. This study highlights that many screen-detected prostate cancers fall into the category of indolent cancer, which may be effectively treated with AS. However, the criteria for subjecting patients to AS and those for intervention in patients already on AS need to be validated.

AS is emerging as a viable strategy for the management of low-risk screen-detected prostate cancer. Dall'Era, *et al*.[1] reported on 321 patients with low-risk PCa managed with AS. Over a median follow-up period of 3.6 years, 243 patients (75.7%) continued on AS, whereas 78 patients underwent delayed active treatment for progression. The disease-specific survival was 100%. Similarly, authors[2] from the Johns Hopkins Institution reported on 407 patients who were managed with AS. Over a median follow-up time of 3.4 years (range: 0.43–12.5 years), 239 (59%) continued on AS and 103 (25%) underwent curative intervention.

These studies indicate an impending paradigm shift in the way early prostate cancer may be managed.
